# Astrocyte-specific hypoxia-inducible factor 1 (HIF-1) does not disrupt the endothelial barrier during hypoxia in vitro

**DOI:** 10.1186/s12987-021-00247-2

**Published:** 2021-03-18

**Authors:** Julia Baumann, Chih-Chieh Tsao, Sheng-Fu Huang, Max Gassmann, Omolara O. Ogunshola

**Affiliations:** 1 Institute of Veterinary Physiology, Vetsuisse Faculty, University of Zurich, Winterthurerstrasse 260, Zurich, Switzerland; 2 Center for Clinical Studies, Vetsuisse Faculty, University of Zurich, 8057 Zurich, Switzerland

**Keywords:** Astrocytes, HIF-1, Endothelial activation, Barrier stability, Primary culture, In vitro BBB

## Abstract

**Background:**

Astrocytes (AC) are essential for brain homeostasis. Much data suggests that AC support and protect the vascular endothelium, but increasing evidence indicates that during injury conditions they may lose their supportive role resulting in endothelial cell activation and BBB disturbance. Understanding the triggers that flip this switch would provide invaluable information for designing new targets to modulate the brain vascular compartment. Hypoxia-inducible factor-1 (HIF-1) has long been assumed to be a culprit for barrier dysfunction as a number of its target genes are potent angiogenic factors. Indeed AC themselves, reservoirs of an array of different growth factors and molecules, are frequently assumed to be the source of such molecules although direct supporting evidence is yet to be published. Being well known reservoirs of HIF-1 dependent angiogenic molecules, we asked if AC HIF-1 dependent paracrine signaling drives brain EC disturbance during hypoxia.

**Methods:**

First we collected conditioned media from control and siRNA-mediated HIF-1 knockdown primary rat AC that had been exposed to normoxic or hypoxic conditions. The conditioned media was then used to culture normoxic and hypoxic (1% O_2_) rat brain microvascular EC (RBE4) for 6 and 24 h. Various activation parameters including migration, proliferation and cell cycling were assessed and compared to untreated controls. In addition, tight junction localization and barrier stability per se (via permeability assay) was evaluated.

**Results:**

AC conditioned media maintained both normoxic and hypoxic EC in a quiescent state by suppressing EC metabolic activity and proliferation. By FACs we observed reduced cell cycling with an increased number of cells in G0 phase and reduced cell numbers in M phase compared to controls. EC migration was also blocked by AC conditioned media and in correlation hypoxic tight junction organization and barrier functionality was improved. Surprisingly however, AC HIF-1 deletion did not impact EC responses or barrier stability during hypoxia.

**Conclusions:**

This study demonstrates that AC HIF-1 dependent paracrine signaling does not contribute to AC modulation of EC barrier function under normoxic or hypoxic conditions. Thus other cell types likely mediate EC permeability in stress scenarios. Our data does however highlight the continuous protective effect of AC on the barrier endothelium. Exploring these protective mechanisms in more detail will provide essential insight into ways to prevent barrier disturbance during injury and disease.

**Supplementary Information:**

The online version contains supplementary material available at 10.1186/s12987-021-00247-2.

## Background

Astrocytes (AC) are key regulators of CNS homeostasis. Being one of the most abundant cell types in the brain they play crucial roles in both brain health and disease [1]. They are essential in regulating cerebral ion homeostasis [2] and give structural and metabolic support to neurons [3, 4]. AC also play a key role in effective functioning of the blood–brain barrier (BBB), and neurovascular unit (NVU) as a whole. The BBB is a critical interface between the circulatory system and the CNS that has a unique vascular architecture. The vessels are composed of specialized brain endothelial cells (EC) that form a restrictive barrier due to high expression of tight junction (TJ) proteins [5] and various transporters and enzymes that selectively facilitate nutrient transport while preventing toxin and pathogen entry [6]. The EC are surrounded by AC end-feet and pericytes, which cover the majority of the abluminal vascular surface and induce and strengthen barrier functionality [7, 8]. As the cellular link between the vascular compartment and brain parenchyma, AC are thought to be a major integration site of brain metabolism and function [9].

Astrocytes are known to secrete a large number of substances including peptides, growth factors and chemokines several of which sustain and/or modulate barrier function [4, 10–13]. They are also highly stress resistant, a feature that facilitates their neuro- and cyto-protective roles. During injury for instance they retain the ability to take up and/or recycle potassium and glutamate as well as release mitogenic and metabolic factors such as ATP that can aid surrounding cells including EC [3, 4]. However injury conditions can cause AC proliferation and retraction of their end-feet from vessel walls, resulting in increased BBB permeability [1, 14]. Additionally, altered molecular and metabolic signaling can also activate barrier EC leading to BBB disturbance [7]. Since an uncompromised and stable BBB is highly dependent on maintenance of a non-activated quiescent EC state, understanding how AC respond to different insults is important to prevent vascular leakage and limit subsequent brain damage.

A key regulator of stress-induced cellular responses, and angiogenesis in general, is hypoxia-inducible factor 1 (HIF-1). HIF-1 is a heterodimer consisting of an unstable oxygen dependent α-subunit and a β-subunit that is permanently expressed in the nucleus [15]. Under normal physiological conditions the α-subunit is constantly degraded but during injury it is stabilized, translocates to the nucleus and dimerizes with the β-subunit. Subsequent co-factor recruitment leads to the formation of the functional protein complex and induction of downstream target genes such as glucose transporter-1 (Glut1), matrix metalloproteinase-9 (MMP9) and vascular endothelial growth factor (VEGF). HIF-1 target genes promote mechanisms that facilitate cellular and organ adaptation to stress conditions [15]. Although HIF-1 mediated vascular remodeling benefits hypoxic or ischemic tissue recovery in non-cerebral vascular beds [16] an increasing number of in vitro and in vivo studies provide convincing evidence that HIF-1 stabilization in the brain induces BBB dysfunction. For example in various rat models of cerebral focal ischemia, pharmacological HIF-1 inhibition reduced infarct volume, BBB permeability and edema formation post stroke [17–20]. Although the HIF-1 target gene VEGF frequently plays a role in such studies, the cellular origin(s) of the negative signals remain largely unidentified. In this regard in vitro studies have provided important insight. Activation of the HIF-1 pathway in iPSC-derived brain EC impaired BBB stability in correlation with a loss of the TJ claudin-5 expression [21]. Similarly, HIF-1 inhibition in rat brain EC prevented hypoxic TJ delocalization and permeability increase [18, 22]. Thus one of the major sources seems to be the endothelium itself. Nevertheless perivascular cells are also likely to play an instrumental role during stress in vivo. As well-known reservoirs of HIF-1 dependent angiogenic molecules, AC in particular could be perpetuators of injury-induced BBB disruption [23, 24]. Indeed, increased AC VEGF levels and secretion has been shown to compromise or correlate with barrier stability in inflammatory models in vivo [25, 26] and in vitro [27]. However, despite being widely assumed the culprit during all scenarios, it is still unclear whether induction of the AC HIF-1 signaling pathway mediates vascular remodeling and BBB disruption.

We therefore asked if hypoxia-induced AC HIF-1-dependent paracrine signaling activates brain EC and drives barrier disturbance. We exposed hypoxic brain EC to conditioned media obtained from control and HIF-1 knockdown (KD) primary rat astrocytes exposed to normoxia or oxygen deprivation. A number of activation parameters, survival as well as barrier stability were measured. Our data convincingly shows that AC conditioned media protects the endothelial barrier in all conditions but surprisingly HIF1 signaling per se had no impact.

## Materials and methods

### Primary astrocyte isolation and cell culture

Primary rat astrocytes were isolated from neonatal pups according to an established protocol [22, 28]. In short, pups were anesthetized by hypothermia, decapitated and the brains removed. The cortical tissue was excised, meninges removed and mechanically dissociated, digested for 12 min at 37 °C. The digestion was stopped with DNase1 (Roche, Switzerland) and trypsin inhibitor (Gibco®, Life Technologies, Switzerland) then triturated. After centrifugation the cell pellet was resuspended in astrocyte media and plated on gelatin-coated petri dishes. AC were cultured in DMEM supplemented with 10 % FBS (Gibco®), 1 % Penicillin/Streptomycin (Gibco®), 2mM l-glutamine (Gibco®) and 50 µg/mL gentamycin sulphate (AppliChem, Germany). Cells were used after first passage. The rat brain endothelial cell line RBE4 [22, 28] was used between passage 36 and 49. Cells were maintained on 250 µg/mL rat-tail collagen coated petri dishes in a 1:1 αMEM/Ham’s F-10 medium mixture (Gibco®) supplemented with 10 % FBS, 300 µg/mL Geneticin (Gibco®) and 1ng/mL basic fibroblast growth factor (Pepro Tech, USA). Rat-tail collagen was isolated as previously described [22, 28]. All cells were cultured in a humidified incubator at 37 °C, 5% CO_2_ and 21% O_2_.

### siRNA transfection

Transfection of 90% confluent primary AC was performed with Oligofectamine™ transfection reagent (Invitrogen, Thermo Fischer Scientific, USA) with HIF-1α targeting siRNA or a non-targeting scrambled siRNA control (100nM, On-TARGETplus, Dharmacon Horizon Discovery, UK). Oligofectamine™ was premixed in Opti-MEM® (Gibco®) and incubated for 10 min before being added to siRNA diluted in Opti-MEM®. After further 20 min incubation at room temperature, the transfection mix was added dropwise to AC in serum free media and incubated for 6 h before replacement with normal culture media. Experiments were subsequently performed after 48 h.

### Hypoxic and conditioned media exposures

**Fig. 1:**
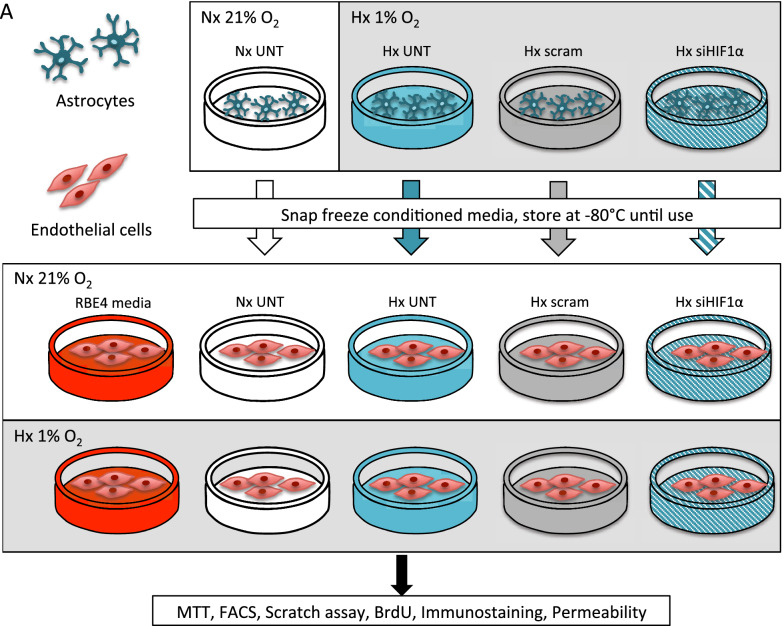
Schematic of AC-CM experiments. Graphical overview of experimental setup using primary AC (dark green) and the RBE4 EC cell line (red). Media was collected from normoxic (white) or hypoxic untreated (UNT, teal), scrambled transfected (scram, grey) or siHIF-1α transfected AC (si-HIF1α, teal-dashed) and snap frozen before use. Thawed AC-CM was transferred to confluent RBE4 immediately before exposure. All experiments were compared to RBE4 grown in their own culture media (RBE4 media, red)

O_2_ deprivation was performed in a hypoxic glove box chamber at 37 °C, 5% CO_2_ and 1% O_2_ (InVivO_2_ 400, Ruskinn Technologies, UK) for 6 h and 24 h. Normoxic controls were maintained at 37 °C, 5% CO_2_ and 21% O_2_. AC-conditioned media (AC-CM) was collected post exposure, immediately snap frozen then stored at − 80 °C until use. On thawing AC-CM was quickly added to cultured RBE4 immediately prior to exposure. A schematic overview of the experimental setup showing conditioned media exchange between primary AC and EC, work flow of media transfer and performed analysis is presented in Fig. 1.

### Cell migration

Migration experiments were performed in duplicate on confluent RBE4 cells grown in 6-well plates. Cells were scratched vertically with a 200 µL pipette tip and three pictures taken per scratch before and after the exposures at marked intervals with an inverted microscope coupled to an 8-bit CCD camera (Axiocam HR, Carl Zeiss, Switzerland). Image analysis to determine scratch width was done using ImageJ software (NIH, USA). Mean migration (µm/h) of the cells was determined by dividing the amount of scratch width closure (µm) by the exposure time (h).

### Cell viability

MTT assay was used to assess viability of confluent cells grown on 96 well plates. After exposures, MTT solution (Sigma-Aldrich, USA) was added to the media (final concentration 0.5 mg/mL) and plates incubated for 1 h at 37 °C. The solution was then removed and formazan crystals dissolved in DMSO (Sigma-Aldrich). All conditions were run in triplicate. Optical density was measured at 570 nm on a spectrophotometer (Thermo Labsystems, Multiskan RC Model 351) with reference filter at 670 nm.

### Western blotting

Cells were washed with ice cold PBS and lysed in whole cell lysis buffer (50 mM Tris, 150 mM NaCl, 1% Triton X-100, 1% NP-40) supplemented with protease inhibitor cocktail (Calbiochem, Germany), 1 mM sodium orthovanadate and 0.5 mM phenylmethansulfonyl fluoride. Protein concentrations were determined with a Pierce BCA protein assay (Thermo Fischer Scientific, USA). Total proteins (30 µg) were separated by SDS-PAGE on 10% gels and transferred onto nitrocellulose membranes (Amersham™ 0.45 μm, Germany). Membranes were blocked with 5% non-fat dried milk dissolved in TBS and incubated at + 4 °C overnight in primary antibodies against HIF-1α (NB100-479 W-1, 1:1000, Novusbio) and β-actin (A5441, 1:5000, Sigma-Aldrich). Membranes were washed with 0.1% Tween-20 TBS then incubated HRP-conjugated secondary antibody in TBS (Jackson ImmunoResearch, UK). Bands were detected with a luminescent image analyzer (Fujifilm, LAS-3000, Switzerland) and a homemade detection solution or the sensitive SuperSignal West Femto Substrate (Thermo Fischer). Quantification was performed with ImageJ software (NIH, USA) using β-actin as loading control.

### Quantitative real-time PCR

RNA was extracted from cell cultures with TRIzol® reagent (Thermo Fisher) according to the manufacturers’ instructions. One µg of total RNA was reverse transcribed using the ImProm-II ReverseTranscriptase kit (Promega, Switzerland) with oligo-dT primers according to the kit protocol. Real-time quantitative PCR (qPCR) was performed using Power SYBR® Green Master Mix (Applied Biosystems, Switzerland) in an ABI 7500 Fast Real-Time PCR System (Applied Biosystems). The following primers (Microsynth AG, Switzerland) were used at 100 nm final concentration: VEGF-A 5′-CGC AAG AAA TCC CGG TTT AA-3′ and 5′-CAA ATG CTT TCT CCG CTC TGA-3′, Glut1 5′-GGG CAT GAT TGG TTC CTT CTC-3′ and 5′-CAG GTT CAT CAT CAG CAT GGA-3′, CA9 5′-CTC TCT CCG TTT CCT TGT GG-3′ and 5′-CCA CTT CTG TGC CTG TGC T-3′, MMP9 5′-TCT GCC TGC ACC ACT AAA GG-3′ and 5′-CAG GCT GTA CCC TTG GTC TG-3′ and β-actin 5′-CTG GCT CCT AGC ACC ATG AAG-3′ and 5′-GCC ACC GAT CCA CAC AGA GT-3′. Ten-fold cDNA dilution series were used to establish optimal primer conditions resulting in > 90% efficiency. To exclude primer dimer and off-target amplifications melting curves were performed and single target amplification confirmed by gel electrophoresis. Experiments were run in duplicate for each condition. All data was normalized to β-actin and fold changes calculated with the ΔΔCt method [29].

### Fluorescence-activated cell sorting (FACS)

Trypsinized cells were harvested from 6-well culture plates, washed in PBS, then fixed in pre-chilled (− 20 °C) 70 % ethanol overnight. After washing in FACS buffer (1mM EDTA, 2% FBS in PBS) the cells were stained with 2 µg/mL Hoechst 33,342 (Sigma-Aldrich) for 20 min at RT before FACS analysis. Sample fluorescence was acquired on a flow cytometer (Gallios 10 C 3 L, Beckman Coulter, USA) equipped with a UV laser for Hoechst excitation. 50,000 cells were measured at medium speed and gated to exclude doublets. Selective separation of G1, S and G2/M cell cycle phases was performed [30] and analysis performed with the Kaluza Analysis program (Beckman Coulter).

### Immunofluorescence

RBE4 cells were seeded on poly-l-lysine (Sigma-Aldrich) and rat-tail collagen coated glass coverslips one day prior to experiments. Post exposure cells were fixed in 4% PFA, permeabilized with 0.1% Triton X-100 in PBS, then blocked with 10% normal goat serum before incubation in ZO-1 primary antibody overnight at 4 °C. Cells were then incubated with Alexa488-conjugated secondary antibody (Thermo Fisher, 1:500), then counterstained with DAPI (Sigma-Aldrich, 100 nM) and coverslips mounted with fluorescent mounting media (Dako, USA). To stain the actin cytoskeleton, fixed and blocked cells were incubated with rhodamine-conjugated phalloidin (Thermo Fisher, 1:10,000) and counterstained with DAPI before being mounted. Images were acquired using a fluorescence microscope coupled to an 8-bit CCD camera (Axiocam HR, Carl Zeiss, Switzerland) and processed using ImageJ software (NIH, USA).

### Permeability assay

RBE4 were grown to confluency on 0.4 μm polycarbonate Transwell inserts (Corning Incorporated, USA), with a blank insert used as an empty filter control. AC-CM was added to both upper and lower compartments of all inserts prior to exposure. After exposure the media was replaced with DMEM (Gibco®) with that in the upper compartment containing 1 mg/mL Lucifer Yellow CH, lithium salt (Invitrogen, Thermo Fischer). Aliquots were taken from the bottom compartment at 15, 30 and 45 min and tracer flux measured with a fluorescence plate reader (FLx800, Biotek Instruments, USA). After subtracting the baseline permeability of the blank insert from the experimental conditions, the permeability coefficient (Pe) was calculated from the clearance slope obtained from measurements at the different time points as previously described [28].

### Statistical analysis

Results are expressed as mean values ± standard deviation of at least 3 independent experiments. Statistical analysis was performed with GraphPad Prism 6 (GraphPad Software, USA) and significance determined by unpaired Student’s t-test with homoscedasticity, or two-way ANOVA for comparison between different exposure groups. Fischer’s LSD post-hoc correction was applied. A p value < 0.05 was considered significant.

## Results

### Confirmation of HIF-1α deletion in primary astrocytes

**Fig. 2:**
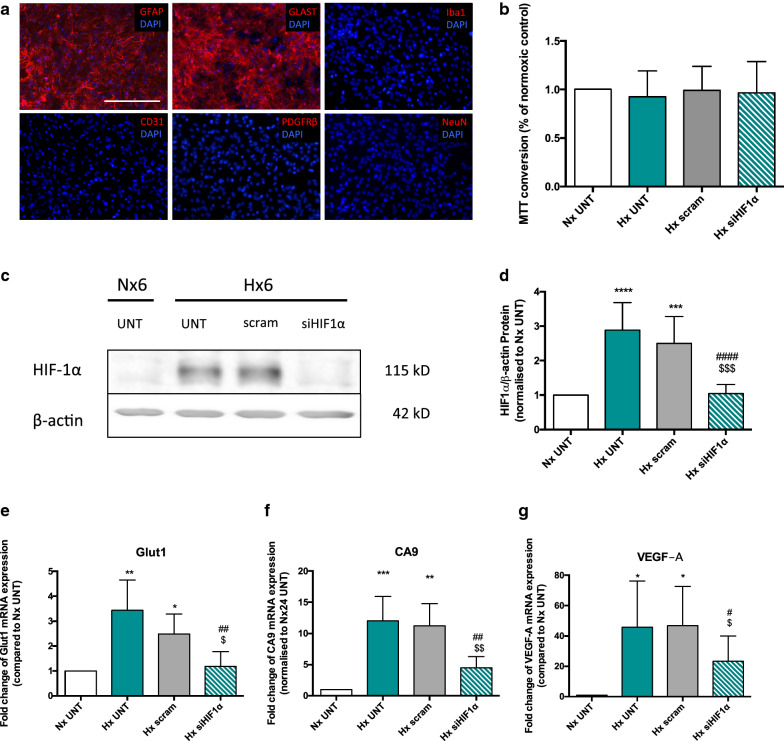
Confirmation of HIF-1α deletion in primary astrocytes. **a** Micrographs showing primary rat astrocytes immunostained for GLAST, GFAP, Iba1, CD31, PDGFRβ and NeuN expression, counterstained with DAPI (blue). Scale bar = 200 nm. **b** MTT conversion of primary AC after 24 h normoxic or hypoxic exposure with HIF-1 KD (siHIF1α) or untreated (UNT) and scrambled controls (scram). **c** Representative Western blot and **d** quantification of HIF-1α KD efficiency in primary ACs after 6 h hypoxia. Messenger RNA levels of HIF-1 target genes Glut1, CA9 and VEGF-A in primary KD ACs compared to normoxic and hypoxic controls after 24 h hypoxia (**e**–**g**). Students t-test and 2way ANOVA, mean ± SD, n = 4–6, *p < 0.05, **p < 0.01, ***p < 0.001, ****p < 0.0001 compared to Nx UNT, ^#^p < 0.05, ^##^p < 0.01 ^####^p < 0.0001 to Hx UNT, ^$^p < 0.05, ^$$$^p < 0.001 compared to Hx scram

To investigate AC HIF-1 dependent paracrine signalling, an in vitro model was established using primary rat astrocytes and the rat brain endothelial cell line RBE4. Purity of AC cultures was > 98 % as controlled by GFAP and GLAST versus Iba1 (microglia), PDGFRß (pericytes) and CD31 (endothelial) expression levels (Fig. 2a). HIF-1 KD astrocytes were generated using siRNA targeting the HIF-1α (siHIF1α) subunit and controlled with non-targeting scrambled (scram) or untreated AC (UNT). Viability was not affected by the transfection procedure (Fig. 2b). Western blot (Fig. 2c) and quantification (Fig. 2d) confirmed hypoxia-induced HIF-1α stabilisation at 6 h was abrogated by up to 95% by siRNA treatment. Decreased mRNA levels of HIF-1 target genes Glut1 (Fig. 2e), CA9 (Fig. 2f) and VEGF-A (Fig. 2g) after 24 h hypoxia confirmed long-term KD efficiency.

### Astrocyte HIF-1 paracrine signalling does not affect endothelial cell metabolic activity and proliferation

**Fig. 3:**
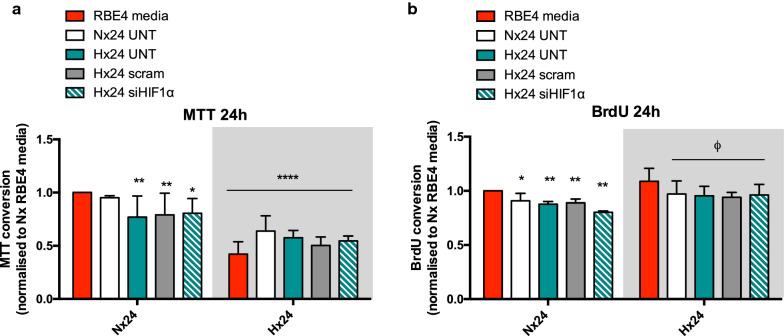
Astrocyte HIF-1 paracrine signalling does not affect endothelial cell metabolic activity and proliferation. Graphical representation of EC **a** mitochondrial activity and **b** proliferation as measured by BrdU incorporation after 24 h exposure. EC were exposed to normoxic or hypoxic conditions with AC-CM or their own RBE4 media. Data is presented in comparison to baseline controls (normoxic RBE4 media control; red bar). 2way ANOVA, mean ± SD, n = 4–6, *p < 0.05, **p < 0.01, ****p < 0.0001 compared to RBE4 media Nx, ^ϕ^p < 0.05 to RBE4 media Hx

Hypoxic EC activation and resulting angiogenic processes, many of which are mediated by HIF-1, can lead to BBB disturbance [7, 31]. Hypoxia-induced BBB disruption could be mediated by AC-derived molecules as the cells have close contact with the barrier endothelium and are known reservoirs of HIF-1 dependent angiogenic factors [23]. We evaluated whether conditioned media (AC-CM) of HIF-1 KD AC alters endothelial quiescence during normoxia and hypoxia, compared to non-KD controls. As designated in Fig. 1, cells were exposed to control EC media or AC-CM before being incubated in normoxia or hypoxia for 6 h or 24 h. AC-CM had no effect on EC normoxic or hypoxic metabolic activity as measured by MTT conversion during 6 h exposures (Additional file 1: Figure S1A). However at 24 h normoxic EC cultured in hypoxic AC-CM media had decreased metabolic activity compared to cells cultured in normoxic AC-CM or RBE4 culture media (Fig. 3a). Interestingly, KD of AC HIF-1 had no effect. At 24 h, hypoxia alone decreased EC metabolic activity but no further suppression was observed with AC-CM (Fig. 3a). AC HIF-1 KD again had no effect on outcome (Fig. 3a). Similarly EC proliferation, as measured by BrdU incorporation, remained unchanged at 6 h time points (Additional file 1: Figure S1B). During 24 h however AC-CM treatment significantly reduced EC proliferation compared to cells held in their own RBE4 media, during normoxia and hypoxia (Fig. 3b). Once again however KD of HIF-1 had no impact EC. Overall these results show that prolonged exposure to particularly hypoxic AC-CM promotes EC quiescence by limiting their metabolic activity and proliferation. Notably, AC HIF-1 induction seems to not be involved in either process.

### AC conditioned media suppresses hypoxia-induced EC cell cycling

**Fig. 4:**
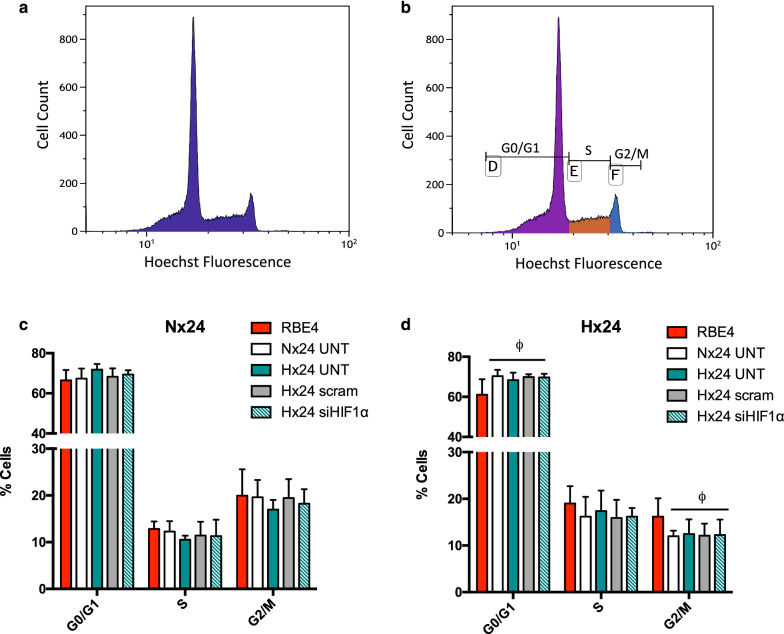
AC conditioned media suppresses hypoxia-induced EC cell cycling. **a** Representative histogram of FACS performed on EC labeled with the DNA dye Hoechst. **b** Example of single cell selective gating during cell cycle phases G0/G1, S and G2/M. Quantification and graphical representation of cell percentages in the individual cell cycle phases during 24 h **c** normoxic and **d** hypoxic exposures with AC-CM. Students t-test, mean ± SD, n = 3–5, ^ϕ^p < 0.05 compared to RBE4 media Hx

EC activation causes the cells to exit the non-dividing quiescent G0/G1 phase and start synthesis (S) and active mitotic G2/M phases [32]. To further understand how AC HIF-1 affects EC activation we evaluated their cell cycle phases after exposure to AC KD and control media by flow cytometry. EC were simultaneously exposed to normoxia and hypoxia for 6 h and 24 h before being harvested, fixed and stained with the nuclei dye Hoechst. Cells in each condition (representative histogram shown in Fig. 4a) were gated using width and area parameters for G0/G1, synthesis S and interphase/mitosis G2/M phases as seen in Fig. 4b. Quantification at 6 h timepoints (Additional file 2: Figure S2A–C) showed similar percentages of cells in the different cycle phases during normoxia and hypoxia (58.49 ± 7.53% vs. 59.80 ± 5.63% in G0/G1, 21.81 ± 1.22% vs. 18.76 ± 1.54% in S and 19.04 ± 7.93% vs. 20.44 ± 4.86% in G2/M respectively) when EC were cultured in their own RBE4 media. Although AC-CM treatment did not alter 6 h normoxic stages during hypoxia trends to increased cell percentages in G0/G1 phase and decreased numbers in G2/M phase were observed. Distinct changes were seen however after prolonged exposure as shown in Table 1. After 24 h in their own culture media a decreased percentage of cells in G0/G1 in the hypoxic group (61.06 ± 7.31) compared to the normoxic cells (66.47 ± 5.19) was observed (Table 1). This correlated with an increase in the cell percentage in S phase (12.47 ± 1.58 vs. 19.01 ± 3.69) indicating hypoxia stimulates brain ECs to exit G0/G1 phase (Table 1). When treated with AC-CM however the percentage of hypoxic cells in G0/G1 was maintained similar to normoxic values (69.22 ± 2.51%) indicating G0/G1 cell cycle arrest (Fig. 4d, e). Furthermore decreased percentages in the mitotic G2/M phase were noted with AC-CM (12.58 ± 1.88%) compared to controls (18.89 ± 3.63%), suggesting less proliferation in line with the BrdU assay results. Taken together, during prolonged hypoxia AC-CM promotes EC G0/G1 cell cycle arrest preventing transition to G2/M thereby retaining the EC in a less activated state. Notably, AC HIF-1 KD had no additional effect suggesting the pathway does not contribute to EC cell cycling.

**Table 1:** FACS data showing EC numbers (%) in G0/G1, S and G2/M after 24 h exposure to AC conditioned media

	Media	G0/G1 (% cells)	S (% cells)	G2/M (% cells)
Normoxia	RBE4 media	66.47 ± 5.19	12.47 ± 1.58	19.96 ± 5.64
	AC-CM Nx24 UNT	67.37 ± 4.98	12.28 ± 2.22	19.60 ± 3.70
	AC-CM Hx24 UNT	71.77 ± 2.83	10.51 ± 0.87	16.97 ± 2.08
	AC-CM Hx24 scram	68.30 ± 4.11	11.44 ± 2.93	19.45 ± 4.05
	AC-CM Hx24 siHIF1α	69.35 ± 2.18	11.30 ± 3.50	18.23 ± 3.11
Hypoxia	RBE4 media	61.06 ± 7.31	19.01 ± 3.69	16.18 ± 3.94
	AC-CM Nx24 UNT	70.31 ± 3.16^ϕ^	16.19 ± 4.23^ϕ^	11.98 ± 1.21^ϕ^
	AC-CM Hx24 UNT	68.37 ± 17.37^ϕ^	17.37 ± 4.39^ϕ^	12.46 ± 3.14^ϕ^
	AC-CM Hx24 scram	69.86 ± 1.42^ϕ^	15.90 ± 3.90^ϕ^	12.13 ± 2.54^ϕ^
	AC-CM Hx24 siHIF1α	69.70 ± 1.76^ϕ^	16.20 ± 1.83^ϕ^	12.25 ± 3.32^ϕ^

Students t-test mean ± SD, n = 3–5, ^ϕ^p < 0.05 compared to RBE4 media Hx

### Preventing AC HIF-1 stabilization does not alter EC migration

**Fig. 5:**
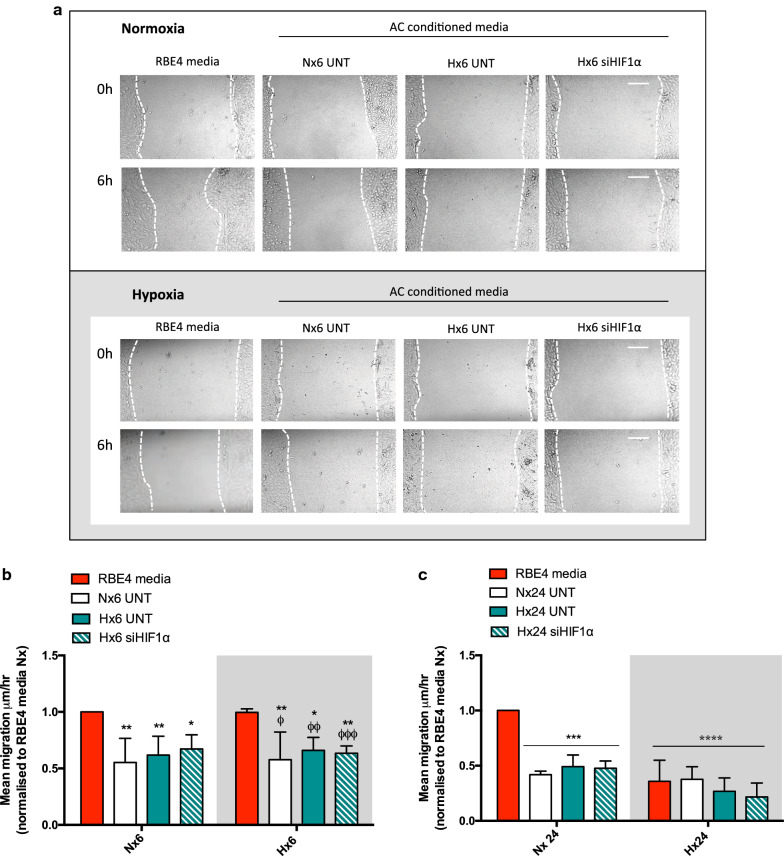
AC HIF-1 KD does not alter EC migration. **a** Representative images of scratched confluent EC monolayers at 0 h and after 6 h exposure. Scale bar = 100 μm. EC were scratched and subsequently treated with RBE4 media or normoxic and hypoxic AC-CM, then exposed to normoxia or hypoxia for 6 h or 24 h. After quantification mean migration is graphed as µm/h for **b** 6 h and **c** 24 h. 2way ANOVA, mean ± SD, n = 3, *p < 0.05, **p < 0.01 ***p < 0.001, ****p < 0.0001 compared to RBE4 media Nx, ^ϕ^p < 0.05, ^ϕϕ^p < 0.01, ^ϕϕϕ^p < 0.001 to RBE4 media Hx

EC migration is a process that underlies increased BBB permeability [7]. We assessed the effect of AC-CM on normoxic and hypoxic EC migration and asked whether AC HIF-1 signaling plays a role using a classical scratch assay. Figure 5a shows representative images of scratched EC monolayers before and after 6 h exposure. Quantification of mean migration (µm/h) shows that at 6 h AC-CM consistently inhibits normoxic and hypoxic EC migration (Fig. 5b). Although at 24 h a similar effect was seen in normoxia, dramatic inhibition of EC migration by hypoxia alone (RBE4 media, red bar) was not further suppressed by AC-CM (Fig. 5c). In all conditions, it was clear that HIF-1 KD did not impact endothelial migration, again agreeing with all other obtained data. Thus AC-driven inhibition of EC migration is HIF-1-independent.

### Protection of barrier functionality by AC-CM is independent of HIF-1

**Fig. 6:**
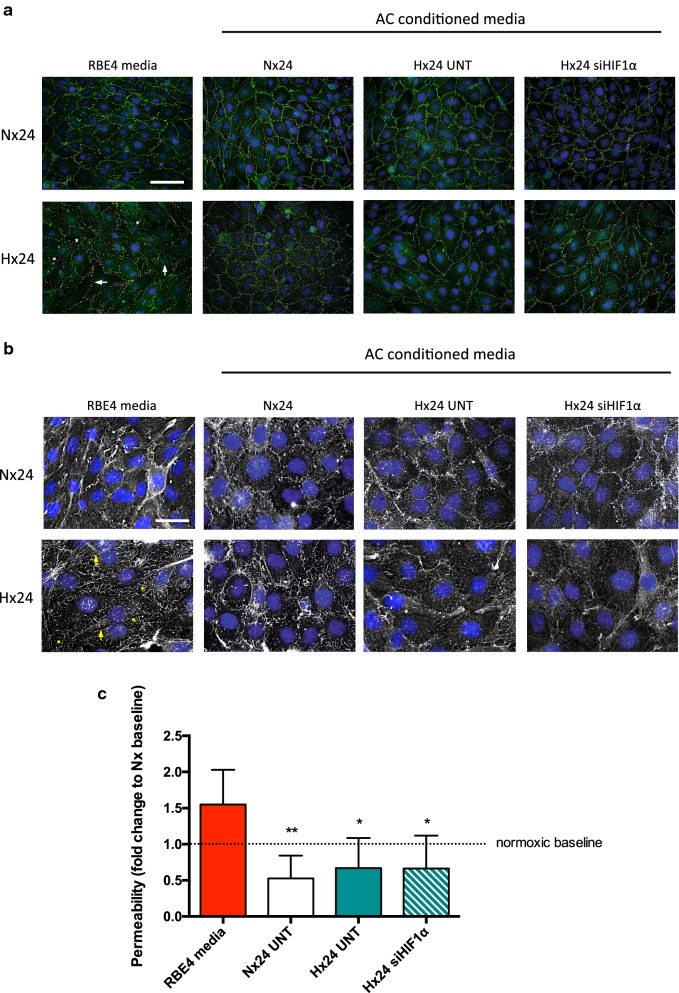
Protection of barrier functionality by AC-CM is independent of HIF-1. **a** Immunostaining of confluent RBE4 labeled for ZO-1 (green) and cell nuclei (DAPI, blue). Cells were cultured in RBE4 or AC conditioned media under normoxia or hypoxia for 24 h. Hypoxia-induced disruption of ZO-1 (arrows) and inter-endothelial gap formation (asterisks) is seen at cell–cell borders. Scale bar = 50 μm. **b** Phalloidin (white) and DAPI stain (blue) of confluent RBE4 monolayers after 24 h normoxic or hypoxic exposure with AC-CM or RBE4 media. Arrows indicate hypoxic stress fiber formation, asterisks highlight inter-endothelial gap formation. Enhanced images, scale bar = 25 μm. **c** Permeability assays were performed on confluent RBE4 on Transwell inserts. Cells were treated with RBE4 media or AC-CM for 24 h. Results are compared to normoxic baseline (dotted line). 2way ANOVA, mean ± SD, n = 3–4, *p < 0.05, **p < 0.01 compared to RBE4 media

Cellular migration requires cytoskeletal rearrangement resulting in delocalization and/or disruption of EC TJ and subsequent BBB impairment [23]. To evaluate barrier functionality we first assessed ZO-1 organization by immunostaining as this key adaptor protein links TJs to the cell cytoskeleton and is delocalized in hypoxic EC in a HIF-1-dependent manner [22]. Representative ZO-1 fluorescent images of EC monolayers after 24 h exposure with AC-CM are presented in Fig. 6. Control normoxic EC display characteristic cobblestone morphology (Fig. 6a) with ZO-1 continuously expressed at cell–cell borders without gap formation, demonstrating tight association between neighbouring cells. Exposure to AC-CM did not alter normoxic ZO-1 localization and the EC monolayer remained intact. As expected hypoxic exposure induced characteristic ZO-1 disruption, inter-endothelial gap formation and cell swelling in monolayers cultured in their own media compared to normoxic controls. Notably, these hypoxic effects were abrogated when ECs were cultured in normoxic AC-CM (Fig. 6a). Furthermore, when cultured in hypoxic AC-CM, despite occurrence of hypoxia-induced cell swelling no gap formation at the cell borders was observed. Indeed, the monolayer remained largely intact and ZO-1 localization was maintained at the cell–cell borders similar to normoxic controls (Fig. 6a). Phalloidin cytoskeleton staining showed normoxic EC display organised F-actin at cell borders and a cortical actin ring demonstrating intact cell contacts within the monolayer (Fig. 6b). In contrast, hypoxia induced clear disorganisation of actin bundles, widespread stress fiber assembly (highlighted by arrows) and inter-endothelial gap formation (asterisks). AC-CM however improved cell contacts and prevented hypoxic gap formation with a more intact cortical actin ring being visible. The improvements were most evident in hypoxic EC treated with normoxic AC-CM (Fig. 6b). The functional consequence of these observations was revealed by in vitro permeability assays. As seen in Fig. 6c, hypoxia-induced permeability occurred as expected compared to the normoxic baseline when EC were cultured in their own media. Importantly, injury-induced permeability was completely abrogated to levels below the normoxic baseline by all AC-CM, with no evident effect of HIF-1 KD. We conclude that astrocyte conditioned media per se suppresses barrier disturbance by maintaining TJ localization thereby fostering intact and tight cell–cell contacts.

## Discussion

It has been long known that AC have growth factor reserves that support and protect the vascular endothelium during stress conditions. More recently however increasing evidence has shown that perivascular cells may lose their supportive roles during injury resulting in activation of the microvascular endothelium and BBB disturbance. Understanding the triggers that flip these switches would provide invaluable information for designing new targets to modulate the brain vascular compartment. As many potent angiogenic factors are HIF-1 target genes, we performed conditioned media experiments to evaluate whether AC HIF-1-mediated paracrine signaling could drive endothelial activation and migration. We consistently observed that AC conditioned media prevented hypoxia-induced EC cell cycling and migration, improved endothelial barrier integrity and prevented hypoxia-induced increases in permeability. Surprisingly however, deletion of AC HIF-1 did not alter injury-induced EC responses or barrier stability during hypoxia suggesting astrocyte-endothelial paracrine signaling is critical for BBB stability but HIF-1 independent.

Vascular homeostasis depends on the balance of pro- and anti-angiogenic factors and is tightly regulated by inter- and intracellular signaling pathways, as well as interactions between cells and the extracellular matrix [7, 32]. Under physiological conditions perivascular cells, both AC and pericytes, support the BBB. During injury conditions however, increased secretion of HIF-1 dependent angiogenic molecules by AC has been suggested to detrimentally modulate the barrier endothelium [25, 33]. Despite successfully blocking HIF-1α stabilization and induction of HIF-1 dependent molecules in AC, no impact on EC activation was observed in this study. Notably, conditioned medium experiments such as this may not wholly capture in vivo paracrine signalling as functional crosstalk between AC and ECs is excluded. Despite this, recent in vivo work from our group also revealed that AC-specific conditional HIF-1 knockout (Gfap/GlastCreER^T2^:Hif1α^fl/fl^) does not abrogate hypoxia-induced BBB disruption (Ogunshola et al. in preparation). A similar observation was seen in a mouse experimental MS model [25]. Interestingly, deletion of the notorious HIF-1 target gene VEGF-A in AC prevented injury-induced BBB disruption and improved outcome in the same study [25] and an in vitro model of Alzheimer disease [34]. As we noted increased VEGF mRNA levels during hypoxic exposure despite HIF-1 blockade, similar to others [35], the data clearly suggests a HIF-1 independent pathway contributes to AC VEGF levels. How the specificity arises that VEGF secreted by AC does not induce angiogenesis is unclear, but different isoforms of VEGF and its receptors clearly exist. In fact, virtually all cells are known to express a VEGF splice variant called VEGF_165_b that exhibits anti-angiogenic properties abrogating normal manifestation of the standard isoform [36]. Whether AC VEGF includes this splice variant and/or an intricate and complex cell-specific distribution exists is yet to be studied. The question then arises why produce angiogenic molecules if not to modulate the brain endothelium? Data clearly shows that VEGF is not only a vasoactive factor but also, similar to HIF-1, cytoprotective and neuroprotective [37]. Indeed endogenous VEGF secretion during injury increased astrocyte survival and proliferation per se [35, 38]. Similarly, protection of the endothelium rather than activation is likely. It is also feasible that multiple astrocyte-derived factors are directed primarily towards neurons and not the endothelium. In line with this notion primary rat AC were shown to polarize VEGF secretion to the extracellular matrix and thus spatially restrict its concentrations [39]. Further research on cell-specific isoforms, direction of secretion and targets of AC paracrine signaling would provide considerable insight.

A critical balancing act of EC quiescence and/or activation is required in all vascular structures. In non-brain vasculature, hypoxic activation of the endothelium can result in the formation of life-saving collateral vessels but also contribute to numerous pathologies [40]. In contrast, in the healthy adult CNS retaining a viable yet angiostatic quiescent EC status ensures an impermeable and stable BBB [41, 42]. Despite using a cell line that is very hypoxia responsive, i.e. as opposed to primary ECs that are much more quiescent, our data underlines the fact that AC conditioned media constantly preserves the barrier functionality. AC are perfect partners being highly adaptable to stress stimuli and able to fulfill protective roles even during severe conditions [4, 43]. Paracrine signaling clearly plays a key role in maintaining this vascular equilibrium as AC-CM consistently prevented hypoxia-induced EC activation by arresting the cells in the G0 cell cycle stage and suppressing injury-mediated BBB permeability. Several other in vitro studies also showed AC co-culture [44–46] or AC-CM [47] improves EC barrier integrity and suppresses proliferation [31]. Anti-migratory effects of AC paracrine signaling on the mouse retinal endothelium [48] and intriguingly on neighbouring Schwann cells [49] have also been reported. It seems likely that many AC-derived factors can protect the barrier endothelium. In in vitro model of CNS inflammation AC secretion of retinoic acid prevented EC oxidative stress and improved BBB despite the inflammatory environment [50]. Secretion of apolipoprotein E [51] and inflammation-associated pentraxin 3 [52] protected the BBB during ischemic injury. A diverse array of metabolites are also increased by AC during injury conditions that can potentially aid EC function [4]. A good example is glutathione, an antioxidant directly shuttled from AC to EC [53] that prevents barrier disturbance during hypoxia and ischemia [53, 54]. Overall however, blocking HIF-1 mediated signaling did not detectably alter AC positive effects.

Some limitations of this study should be acknowledged. The work was conducted with an in vitro model of the BBB and such models are known to harbour some limitations in relation to the in vivo situation. As our experimental design utilized transfer of conditioned AC media to EC, any additional influence of endothelial-derived stress signals and/or crosstalk between the cells on the AC secretory response is not elucidated herein. We also cannot exclude that exposing the cells to a more significant insult such as oxygen glucose deprivation (ischemia) might result in a different outcome. Future co-culture experiments will provide more insight.

## Conclusions

In conclusion, this study demonstrates that AC HIF-1 dependent paracrine signaling does not disrupt the endothelial barrier during hypoxia. It does however highlight the incessant protective effect of AC on the barrier endothelium. Exploring mechanisms of AC protection and especially the factors they secrete in more detail will provide essential insight on ways to support the BBB during injury and neurological disease.

## Supplementary Information

**Additional file 1: Figure S1.** AC conditioned media does not alter EC metabolic activity or proliferation at 6 h. (A) Graphical representation of EC mitochondrial activity as measured by MTT after 6 h normoxic or hypoxic exposure, with AC-CM or RBE4 media control. (B) Proliferation measured by BrdU incorporation was performed after 6 h normoxia or hypoxia. Mean ± SD mean n = 4–6.

**Additional file 2: Figure S2.** AC conditioned media does not affect 6 h EC cell cycling. Quantification and graphical representation of cell percentages in the individual cell cycle phases during 6 h (A) normoxic and (B) hypoxic exposures with AC-CM. Mean ± SD mean n = 4. (C) FACS analysis of numbers (%) of EC in G0/G1, S and G2/M phases after 6 h exposure to AC-CM. Students t-test, mean ± SD, n = 3.

## Data Availability

The datasets used and/or analysed during the current study are available from the corresponding author on reasonable request.

## Abbreviations

AC: Astrocytes
AC-CM: Astrocyte conditioned medium
BBB: Blood–brain barrier
BCA: Bicinchoninic acid
bFGF: Basic fibroblast growth factor
CA9: Carbonic anhydrase 9
DMEM: Dulbecco’s Modified Eagle’s medium
EC: Endothelial cells
FACS: Fluorescence-activated cell sorting
GLUT1: Glucose transporter 1
HIF-1: Hypoxia-inducible factor 1
MMP9: Matrix metalloproteinase 9
PFA: Paraformaldehyde
TGFβ: Transforming growth factor β
SDS-PAGE: Sodium dodecyl sulfate polyacrylamide gel electrophoresis
TBS: Tris-buffered saline
TJ: Tight junction
VEGF: Vascular endothelial growth factor
ZO-1: Zonula occludens 1

## References

[1] Sofroniew MV, Vinters HV (2010). Astrocytes: biology and pathology. Acta Neuropathol.

[2] Hertz L, Chen Y (2016). Importance of astrocytes for potassium ion (K(+)) homeostasis in brain and glial effects of K(+) and its transporters on learning. Neurosci Biobehav Rev.

[3] Ioannou MS, Jackson J, Sheu SH, Chang CL, Weigel AV, Liu H (2019). Neuron-astrocyte metabolic coupling protects against activity-induced fatty acid toxicity. Cell.

[4] Huang SF, Fischer S, Koshkin A, Laczko E, Fischer D, Ogunshola OO (2020). Cell-specific metabolomic responses to injury: novel insights into blood–brain barrier modulation. Sci Rep.

[5] McCaffrey G, Staatz WD, Quigley CA, Nametz N, Seelbach MJ, Campos CR (2007). Tight junctions contain oligomeric protein assembly critical for maintaining blood–brain barrier integrity in vivo. J Neurochem.

[6] Roberts LM, Black DS, Raman C, Woodford K, Zhou M, Haggerty JE (2008). Subcellular localization of transporters along the rat blood–brain barrier and blood-cerebral-spinal fluid barrier by in vivo biotinylation. Neuroscience.

[7] Engelhardt S, Patkar S, Ogunshola OO (2014). Cell-specific blood–brain barrier regulation in health and disease: a focus on hypoxia. Br J Pharmacol.

[8] Janzer RC, Raff MC (1987). Astrocytes induce blood–brain barrier properties in endothelial cells. Nature.

[9] Wolburg H, Noell S, Mack A, Wolburg-Buchholz K, Fallier-Becker P (2009). Brain endothelial cells and the glio-vascular complex. Cell Tissue Res.

[10] Nico B, Ribatti D (2012). Morphofunctional aspects of the blood–brain barrier. Curr Drug Metab.

[11] Shimizu F, Sano Y, Saito K, Abe MA, Maeda T, Haruki H (2012). Pericyte-derived glial cell line-derived neurotrophic factor increase the expression of Claudin-5 in the blood–brain barrier and the blood–nerve barrier. Neurochem Res.

[12] Dohgu ST, Yamauchi F, Nakagawa A, Egawa S, Naito T, Tsuruo M, Sawada T, Niwa Y, Kataoka M (2005). Brain pericytes contribute to the induction and up-regulation of blood–brain barrier functions through transforming growth factor-beta production. Brain Res.

[13] Walshe TE, Saint-Geniez M, Maharaj ASR, Sekiyama E, Maldonado AE, D’Amore PA (2009). TGF-beta is required for vascular barrier function, endothelial survival and homeostasis of the adult microvasculature. PLoS ONE.

[14] Heithoff BP, George KK, Phares AN, Zuidhoek IA, Munoz-Ballester C, Robel S (2020). Astrocytes are necessary for blood–brain barrier maintenance in the adult mouse brain. Glia.

[15] Pugh CW, Ratcliffe PJ (2003). Regulation of angiogenesis by hypoxia: role of the HIF system. Nat Med.

[16] Semenza GL (2014). Hypoxia-inducible factor 1 and cardiovascular disease. Annu Rev Physiol.

[17] Chen W, Jadhav V, Tang J, Zhang JH (2008). HIF-1alpha inhibition ameliorates neonatal brain injury in a rat pup hypoxic-ischemic model. Neurobiol Dis.

[18] Yeh WL, Lu DY, Lin CJ, Liou HC, Fu WM (2007). Inhibition of hypoxia-induced increase of blood–brain barrier permeability by YC-1 through the antagonism of HIF-1alpha accumulation and VEGF expression. Mol Pharmacol.

[19] Chen RL, Ogunshola OO, Yeoh KK, Jani A, Papadakis M, Nagel S (2014). HIF prolyl hydroxylase inhibiton prior to transient focal cerebral ischaemia is neuroprotective in mice. J Neurochem.

[20] Chen CH, Hu Q, Yan JH, Yang XM, Shi XZ, Lei JL (2009). Early inhibition of HIF-1 alpha with small interfering RNA reduces ischemic-reperfused brain injury in rats. Neurobiol Dis.

[21] Page S, Raut S, Al-Ahmad A (2019). Oxygen-glucose deprivation/reoxygenation-induced barrier disruption at the human blood–brain barrier is partially mediated through the HIF-1 pathway. Neuromol Med.

[22] Engelhardt S, Al-Ahmad AJ, Gassmann M, Ogunshola OO (2014). Hypoxia selectively disrupts brain microvascular endothelial tight junction complexes through a hypoxia-inducible factor-1 (HIF-1) dependent mechanism. J Cell Physiol.

[23] Abbott NJ, Ronnback L, Hansson E (2006). Astrocyte-endothelial interactions at the blood–brain barrier. Nat Rev Neurosci.

[24] Min H, Hong J, Cho IH, Jang YH, Lee H, Kim D (2015). TLR2-induced astrocyte MMP9 activation compromises the blood brain barrier and exacerbates intracerebral hemorrhage in animal models. Mol Brain.

[25] Argaw AT, Asp L, Zhang J, Navrazhina K, Pham T, Mariani JN (2012). Astrocyte-derived VEGF-A drives blood–brain barrier disruption in CNS inflammatory disease. J Clin Invest.

[26] Chapouly C, Tadesse Argaw A, Horng S, Castro K, Zhang J, Asp L (2015). Astrocytic TYMP and VEGFA drive blood–brain barrier opening in inflammatory central nervous system lesions. Brain.

[27] Lu DY, Yu WH, Yeh WL, Tang CH, Leung YM, Wong KL (2009). Hypoxia-induced matrix metalloproteinase-13 expression in astrocytes enhances permeability of brain endothelial cells. J Cell Physiol.

[28] Al Ahmad A, Gassmann M, Ogunshola OO (2009). Maintaining blood–brain barrier integrity: pericytes perform better than astrocytes during prolonged oxygen deprivation. J Cell Physiol.

[29] Rao X, Huang X, Zhou Z, Lin X (2013). An improvement of the 2^(-delta delta CT) method for quantitative real-time polymerase chain reaction data analysis. Biostat Bioinforma Biomath.

[30] Darzynkiewicz Z, Huang X (2004). Analysis of cellular DNA content by flow cytometry. Curr Protoc Immunol.

[31] Al Ahmad A, Taboada CB, Gassmann M, Ogunshola OO (2011). Astrocytes and pericytes differentially modulate blood–brain barrier characteristics during development and hypoxic insult. J Cereb Blood Flow Metab.

[32] Zecchin A, Kalucka J, Dubois C, Carmeliet P (2017). How endothelial cells adapt their metabolism to form vessels in tumors. Front Immunol.

[33] Kaur C, Sivakumar V, Zhang Y, Ling EA (2006). Hypoxia-induced astrocytic reaction and increased vascular permeability in the rat cerebellum. Glia.

[34] Spampinato SF, Merlo S, Sano Y, Kanda T, Sortino MA (2017). Astrocytes contribute to Abeta-induced blood–brain barrier damage through activation of endothelial MMP9. J Neurochem.

[35] Schmid-Brunclik N, Burgi-Taboada C, Antoniou X, Gassmann M, Ogunshola OO (2008). Astrocyte responses to injury: VEGF simultaneously modulates cell death and proliferation. Am J Physiol Regul Integr Comp Physiol.

[36] Woolard J, Wang WY, Bevan HS, Qiu Y, Morbidelli L, Pritchard-Jones RO (2004). VEGF(165)b, an inhibitory vascular endothelial growth factor splice variant: mechanism of action, in vivo effect on angiogenesis and endogenous protein expression. Cancer Res.

[37] Shim JW, Madsen JR (2018). VEGF signaling in neurological disorders. Int J Mol Sci.

[38] Krum JM, Khaibullina A (2003). Inhibition of endogenous VEGF impedes revascularization and astroglial proliferation: roles for VEGF in brain repair. Exp Neurol.

[39] Egervari K, Potter G, Guzman-Hernandez ML, Salmon P, Soto-Ribeiro M, Kastberger B (2016). Astrocytes spatially restrict VEGF signaling by polarized secretion and incorporation of VEGF into the actively assembling extracellular matrix. Glia.

[40] Wong BW, Marsch E, Treps L, Baes M, Carmeliet P (2017). Endothelial cell metabolism in health and disease: impact of hypoxia. EMBO J.

[41] Rajani RM, Quick S, Ruigrok SR, Graham D, Harris SE, Verhaaren BFJ (2018). Reversal of endothelial dysfunction reduces white matter vulnerability in cerebral small vessel disease in rats. Sci Transl Med.

[42] Munji RN, Soung AL, Weiner GA, Sohet F, Semple BD, Trivedi A (2019). Profiling the mouse brain endothelial transcriptome in health and disease models reveals a core blood–brain barrier dysfunction module. Nat Neurosci.

[43] Gurer G, Gursoy-Ozdemir Y, Erdemli E, Can A, Dalkara T (2009). Astrocytes are more resistant to focal cerebral ischemia than neurons and die by a delayed necrosis. Brain Pathol.

[44] Fischer S, Wobben M, Kleinstuck J, Renz D, Schaper W (2000). Effect of astroglial cells on hypoxia-induced permeability in PBMEC cells. Am J Physiol Cell Physiol.

[45] Chow J, Ogunshola O, Fan SY, Li Y, Ment LR, Madri JA (2001). Astrocyte-derived VEGF mediates survival and tube stabilization of hypoxic brain microvascular endothelial cells in vitro. Brain Res Dev Brain Res.

[46] Sobue K, Yamamoto N, Yoneda K, Hodgson ME, Yamashiro K, Tsuruoka N (1999). Induction of blood–brain barrier properties in immortalized bovine brain endothelial cells by astrocytic factors. Neurosci Res.

[47] Yamagata K, Tagami M, Nara Y, Mitani M, Kubota A, Fujino H (1997). Astrocyte-conditioned medium induces blood–brain barrier properties in endothelial cells. Clin Exp Pharmacol Physiol.

[48] Hajrasouliha AR, Jiang G, Lu Q, Lu H, Kaplan HJ, Zhang HG (2013). Exosomes from retinal astrocytes contain antiangiogenic components that inhibit laser-induced choroidal neovascularization. J Biol Chem.

[49] Afshari FT, Kwok JC, White L, Fawcett JW (2010). Schwann cell migration is integrin-dependent and inhibited by astrocyte-produced aggrecan. Glia.

[50] Mizee MR, Nijland PG, van der Pol SM, Drexhage JA, van Het Hof B, Mebius R (2014). Astrocyte-derived retinoic acid: a novel regulator of blood–brain barrier function in multiple sclerosis. Acta Neuropathol.

[51] Xiang J, Zhu W, Yang F, Yu ZH, Cai M, Li XT (2020). Melatonin-induced ApoE expression in mouse astrocytes protects endothelial cells from OGD-R induced injuries. Transl Psychiatry.

[52] Shindo A, Maki T, Mandeville ET, Liang AC, Egawa N, Itoh K (2016). Astrocyte-derived pentraxin 3 supports blood–brain barrier integrity under acute phase of stroke. Stroke.

[53] Huang SF, Othman A, Koshkin A, Fischer S, Fischer D, Zamboni N (2020). Astrocyte glutathione maintains endothelial barrier stability. Redox Biol.

[54] Song J, Kang SM, Lee WT, Park KA, Lee KM, Lee JE (2014). Glutathione protects brain endothelial cells from hydrogen peroxide-induced oxidative stress by increasing nrf2 expression. Exp Neurobiol.

