# Supplementary material for: Astrocyte-specific hypoxia-inducible factor 1 (HIF-1) does not disrupt the endothelial barrier during hypoxia in vitro
Source: Fluids Barriers CNS. 2021 Mar 18;18:13. doi: 10.1186/s12987-021-00247-2 (PMC7977259; doi:10.1186/s12987-021-00247-2)

Additional file 1 : Figure S1
AC conditioned media does not alter EC metabolic activity or proliferation at 6h

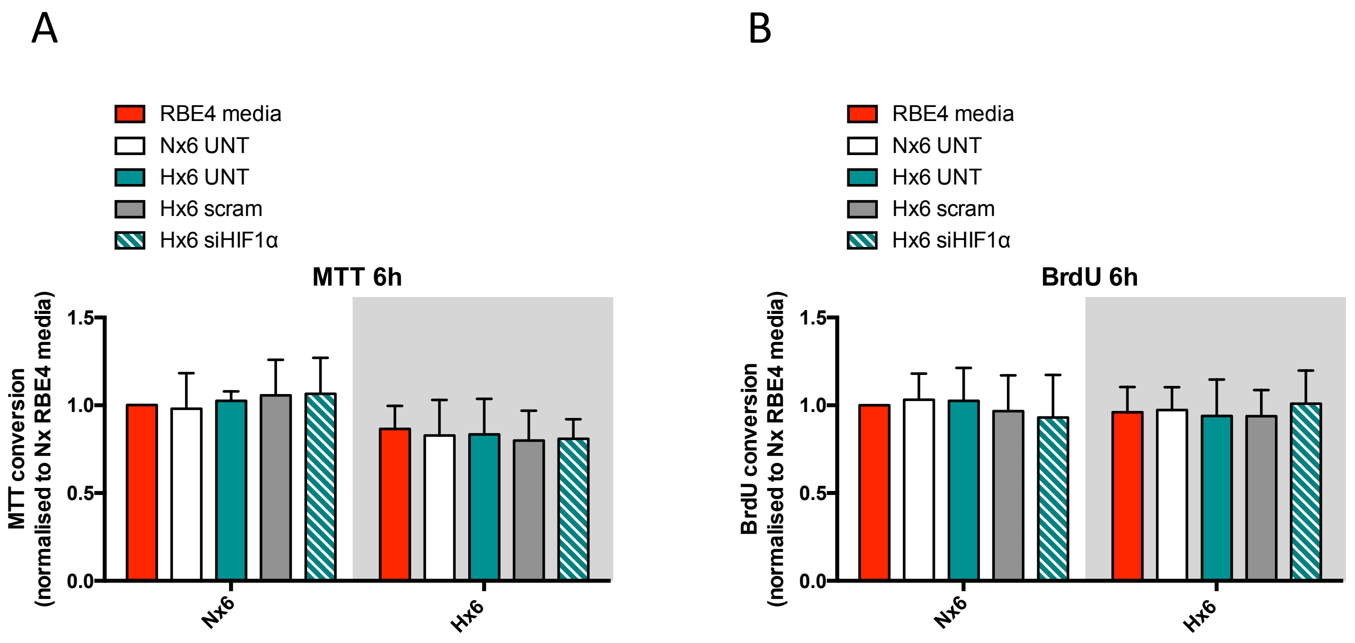

Supplement: Supplementary file 1 — Additional file 1: Figure S1. AC conditioned media does not alter EC metabolic activity or proliferation at 6 h. (A) Graphical representation of EC mitochondrial activity as measured by MTT after 6 h normoxic or hypoxic exposure, with AC-CM or RBE4 media control. (B) Proliferation measured by BrdU incorporation was performed after 6 h normoxia or hypoxia. Mean ± SD mean n = 4–6. [file 12987_2021_247_MOESM1_ESM.pdf]
