# Supplementary material for: Astrocyte-specific hypoxia-inducible factor 1 (HIF-1) does not disrupt the endothelial barrier during hypoxia in vitro
Source: Fluids Barriers CNS. 2021 Mar 18;18:13. doi: 10.1186/s12987-021-00247-2 (PMC7977259; doi:10.1186/s12987-021-00247-2)
Supplement: Supplementary file 2 — Additional file 2: Figure S2. AC conditioned media does not affect 6 h EC cell cycling. Quantification and graphical representation of cell percentages in the individual cell cycle phases during 6 h (A) normoxic and (B) hypoxic exposures with AC-CM. Mean ± SD mean n = 4. (C) FACS analysis of numbers (%) of EC in G0/G1, S and G2/M phases after 6 h exposure to AC-CM. Students t-test, mean ± SD, n = 3. [file 12987_2021_247_MOESM2_ESM.pdf]

Additional file 2 : Figure S2
AC conditioned media does not affect 6h EC cell cycling

A

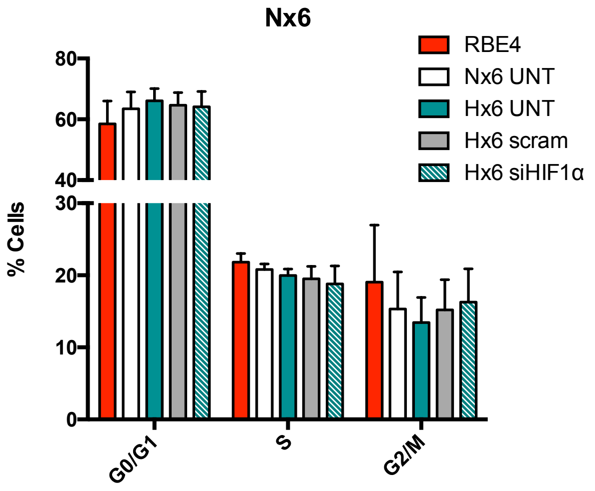

B

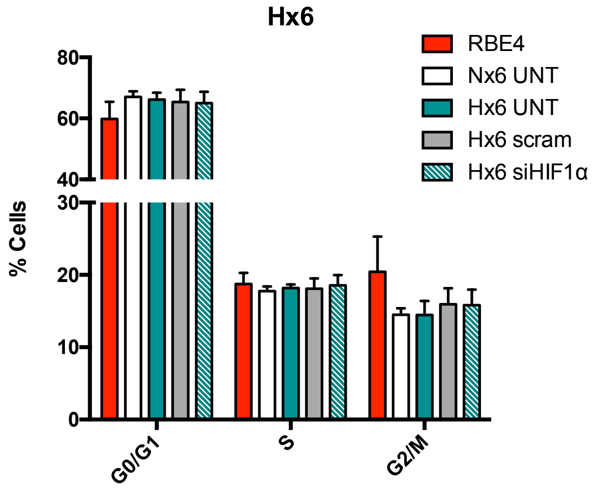

C

	Media	G0/G1 (% cells)	S (% cells)	G2/M (% cells)
Normoxia	RBE4	58.49 ± 7.53	21.81 ± 1.22	19.04 ± 7.93
	AC-CM Nx24 UNT	63.49 ± 5.54	20.80 ± 0.76	15.33 ± 5.14
	AC-CM Hx24 UNT	66.09 ± 4.04	19.97 ± 0.92	13.43 ± 3.49
	AC-CM Hx24 scram	64.67 ± 4.16	19.51 ± 1.75	15.21 ± 4.18
	AC-CM Hx24 siHIF1α	64.09 ± 5.05	18.82 ± 2.48	16.29 ± 4.62
Hypoxia	RBE4	59.80 ± 5.63	18.76 ± 1.54	20.44 ± 4.86
	AC-CM Nx24 UNT	67.07 ± 1.79	17.76 ± 0.65	14.49 ± 0.90
	AC-CM Hx24 UNT	66.14 ± 2.32	18.73 ± 0.52	14.44 ± 1.95
	AC-CM Hx24 scram	65.40 ± 3.95	18.09 ± 1.41	15.93 ± 2.21
	AC-CM Hx24 siHIF1α	64.99 ± 3.69	18.55 ± 1.41	15.82 ± 2.16
